# Supplementary material for: MouseBytes, an open-access high-throughput pipeline and database for rodent touchscreen-based cognitive assessment
Source: eLife. 2019 Dec 11;8:e49630. doi: 10.7554/eLife.49630 (PMC6934379; doi:10.7554/eLife.49630)
Supplement: Transparent reporting form [file elife-49630-transrepform.pdf]

1st Floor
24 Hills Road
Cambridge CB2 1JP, UK

P 01223 855340
W elifesciences.org
T @elife

eLife's transparent reporting form

We encourage authors to provide detailed information *within their submission* to facilitate the interpretation and replication of experiments. Authors can upload supporting documentation to indicate the use of appropriate reporting guidelines for health-related research (see EQUATOR Network), life science research (see the BioSharing Information Resource), or the ARRIVE guidelines for reporting work involving animal research. Where applicable, authors should refer to any relevant reporting standards documents in this form.

If you have any questions, please consult our Journal Policies and/or contact us: editorial@elifesciences.org.

Sample-size estimation

- You should state whether an appropriate sample size was computed when the study was being designed
- You should state the statistical method of sample size computation and any required assumptions
- If no explicit power analysis was used, you should describe how you decided what sample (replicate) size (number) to use

Please outline where this information can be found within the submission (e.g., sections or figure legends), or explain why this information doesn't apply to your submission:

The sample size was based on previous studies using the Bussey-Saksida touchscreen technology. Please see Material and Methods (**Experimental Model and Subject Details – Animals – page 24**). The N value for each group can be found in Supplementary table 1.

Replicates

- You should report how often each experiment was performed
- You should include a definition of biological versus technical replication
- The data obtained should be provided and sufficient information should be provided to indicate the number of independent biological and/or technical replicates
- If you encountered any outliers, you should describe how these were handled
- Criteria for exclusion/inclusion of data should be clearly stated
- High-throughput sequence data should be uploaded before submission, with a private link for reviewers provided (these are available from both GEO and ArrayExpress)

Please outline where this information can be found within the submission (e.g., sections or figure legends), or explain why this information doesn't apply to your submission:

eLIFE
1st Floor
24 Hills Road
Cambridge CB2 1JP, UK
P 01223 855340
W elifesciences.org
T @elife

Each group of mice was tested longitudinally in three distinct touchscreen tests. For the attention test, experiments in males and females were repeated in two different sites. For other touchscreen tests males were tested at the University of Western Ontario and females were tested at the University of Guelph. The MouseBytes quality control system was used to identify and exclude any potential outliers. See **QUANTIFICATION AND STATISTCAL ANALYSIS – Touchscreen data analysis, quality control and storage** for additional information (page 39). The QC and statistics R codes are also provided - https://github.com/srmemar/Mousebytes-An-open-access-high-throughput-pipeline-and-database-for-rodent-touchscreen-based-data

eLife Sciences Publications, Ltd is a limited liability non-profit non-stock corporation incorporated in the State of Delaware, USA, with company number 5030732, and is registered in the UK with company number FC030576 and branch number BR015634 at the address 1st Floor, 24 Hills Road, Cambridge CB2 1JP | August 2014

Statistical reporting

- Statistical analysis methods should be described and justified
- Raw data should be presented in figures whenever informative to do so (typically when N per group is less than 10)
- For each experiment, you should identify the statistical tests used, exact values of N, definitions of center, methods of multiple test correction, and dispersion and precision measures (e.g., mean, median, SD, SEM, confidence intervals; and, for the major substantive results, a measure of effect size (e.g., Pearson's r, Cohen's d)
- Report exact p-values wherever possible alongside the summary statistics and 95% confidence intervals. These should be reported for all key questions and not only when the p-value is less than 0.05.

Please outline where this information can be found within the submission (e.g., sections or figure legends), or explain why this information doesn't apply to your submission:

Descriptions of our statistical analyses, quality control systems, and data processing are described in **QUANTIFICATION AND STATISTICAL ANALYSIS – Touchscreen data analysis**, quality control and storage (page 39). Supplementary Tables contain exact p-values and partial eta-squared values for all primary analyses.

(For large datasets, or papers with a very large number of statistical tests, you may upload a single table file with tests, Ns, etc., with reference to sections in the manuscript.)

Group allocation

- Indicate how samples were allocated into experimental groups (in the case of clinical studies, please specify allocation to treatment method); if randomization was used, please also state if restricted randomization was applied
- Indicate if masking was used during group allocation, data collection and/or data analysis

Please outline where this information can be found within the submission (e.g., sections or figure legends), or explain why this information doesn't apply to your submission:

Group assignment was based on the sex and genotype of the mice obtained from Jax laboratories. See **EXPERIMENTAL MODEL AND SUBJECT DETAILS – ANIMALS** (Page23)

Additional data files ("source data")

- We encourage you to upload relevant additional data files, such as numerical data that are represented as a graph in a figure, or as a summary table
- Where provided, these should be in the most useful format, and they can be uploaded as "Source data" files linked to a main figure or table
- Include model definition files including the full list of parameters used
- Include code used for data analysis (e.g., R, MatLab)
- Avoid stating that data files are "available upon request"

Please indicate the figures or tables for which source data files have been provided:

eLife Sciences Publications, Ltd is a limited liability non-profit non-stock corporation incorporated in the State of Delaware, USA, with company number 5030732, and is registered in the UK with company number FC030576 and branch number BR015634 at the address 1st Floor, 24 Hills Road, Cambridge CB2 1JP | August 2014

Supplementary table 1 – includes the N value for each age group
Supplementary tables 2, 3 and 4 - include the split-plot ANOVA analysis using AGE/Stimulus Duration/ Research Site/ Genotype/Sex as factors
Supplementary tables 5, 6 and 7 – include the split-plot ANOVA analysis using Stimulus duration/Genotype/Sex as factors.
Supplementary table 8 – complete breakdown of all the vigilance analysis using trials blocks/Genotype/Stimulus duration/Sex/Age as factors.
Supplementary table 9 - Gait analysis for 5xFAD mice
All the raw data touchscreen testing for the tables and figures can be found in www.mousebytes.ca
The statistical analysis is described in the Material and Methods – **QUANTIFICATION AND STATITICAL ANALYSIS – Touchscreen data analysis, quality control and storage (Page 39)**. The R codes for the statistical analysis are also provided and can be downloaded - https://github.com/srmemar/Mousebytes-An-open-access-high-throughput-pipeline-and-database-for-rodent-touchscreen-based-data
